# Highly Selective Targeting of Pancreatic Cancer in the Liver with a Near-Infrared Anti-MUC5AC Probe in a PDOX Mouse Model: A Proof-of-Concept Study

**DOI:** 10.3390/jpm13050857

**Published:** 2023-05-20

**Authors:** Michael A. Turner, Kristin E. Cox, Nicholas Neel, Siamak Amirfakhri, Hiroto Nishino, Bryan M. Clary, Mojgan Hosseini, Gopalakrishnan Natarajan, Kavita Mallya, Aaron M. Mohs, Robert M. Hoffman, Surinder K. Batra, Michael Bouvet

**Affiliations:** 1 Division of Surgical Oncology, Department of Surgery, University of California San Diego, La Jolla, CA 92037, USA; 2 VA San Diego Healthcare System, La Jolla, CA 92161, USA; 3 Department of Pathology, University of California San Diego, La Jolla, CA 92037, USA; 4 Department of Biochemistry, University of Nebraska Medical Center, Omaha, NE 68198, USA; 5 Department of Pharmaceutical Sciences, University of Nebraska Medical Center, Omaha, NE 68198, USA; 6 Fred and Pamela Buffet Cancer Center, University of Nebraska Medical Center, Omaha, NE 68105, USA; 7 AntiCancer, Inc., San Diego, CA 92111, USA

**Keywords:** pancreatic cancer, liver metastasis, selective targeting, fluorescent antibody, imaging, mucin

## Abstract

Accurately identifying metastatic disease is critical to directing the appropriate treatment in pancreatic cancer. Mucin 5AC is overexpressed in pancreatic cancer but absent in normal pancreas tissue. The present proof-of-concept study demonstrates the efficacy of an anti-mucin 5AC antibody conjugated to an IR800 dye (MUC5AC-IR800) to preferentially label a liver metastasis of pancreatic cancer (*Panc Met*) in a unique patient-derived orthotopic xenograft (PDOX) model. In orthotopic models, the mean tumor to background ratio was 1.787 (SD ± 0.336), and immunohistochemistry confirmed the expression of MUC5AC within tumor cells. MUC5AC-IR800 provides distinct visualization of pancreatic cancer liver metastasis in a PDOX mouse model, demonstrating its potential utility in staging laparoscopy and fluorescence-guided surgery.

## 1. Introduction

The 5-year overall survival of pancreatic ductal adenocarcinoma (PDAC) is less than 10% [1]. Complete surgical resection remains the only possibility for a cure. However, ~55% of the time, curative resection is not possible due to metastatic disease [2]. For patients without radiographic evidence of metastasis, locally advanced or metastatic disease is found during staging laparoscopy at rates of 14–33% [3,4,5]. The most common sites for metastasis in pancreatic cancer are the liver (41%), peritoneum (11%), and lungs (9%) [2]. There is a critical need for imaging modalities to improve the visualization of metastatic disease and to direct the appropriate treatments. The use of fluorescence imaging could aid in this process. Our laboratory has demonstrated the ability to use cancer-specific antibodies tagged with near-infrared (NIR) fluorophores to target colorectal [6,7,8], gastric [9], and pancreatic [10,11] cancer.

Mucins, a family of glycoproteins, are a potential molecular target for labeling pancreatic cancer. Mucin 5AC (MUC5AC) is an extracellular gel-secreting protein normally found in the stomach, gallbladder, and the lumen of the bronchial and gastrointestinal (GI) tract but absent from normal pancreatic tissue as well as the liver or the serosal surfaces of the GI tract [12,13]. However, MUC5AC expression is found in pancreatic cancer and early pancreatic ductal lesions [14]. Therefore, an anti-MUC5AC fluorescent antibody is an excellent candidate for targeting metastatic pancreatic tumor deposits in the abdomen. The present study reports the use of fluorescent anti-MUC5AC antibody to selectively and brightly label a liver metastasis of pancreatic cancer in a patient-derived orthotopic xenograft (PDOX) mouse model.

Previously, we orthotopically implanted a patient-derived primary pancreatic cancer (*AA1305*) in the pancreas of nude mice to establish a primary pancreatic cancer PDOX model, which was brightly targeted by fluorescent anti-mucin5AC [11]. In the present study, a rare patient-derived liver metastasis of pancreatic cancer was obtained (*Panc Met*) and implanted in the liver of nude mice to establish a pancreatic cancer liver metastasis PDOX model. Here, we show that anti-mucin 5AC selectively and brightly labeled this liver metastasis. The implications of this work for clinical applications in staging laparoscopy and florescence-guided surgery for liver metastases of pancreatic cancer are discussed.

## 2. Materials and Methods

*Mouse Models:* Athymic nude mice, aged 4–6 weeks, were purchased from The Jackson Laboratory (Bar Harbor, ME, USA). They were housed in a barrier facility and fed an autoclaved diet. Prior to any surgical procedure, the mice were anesthetized with a solution of xylazine, ketamine, and phosphate-buffered saline (PBS) via intraperitoneal injection. At the conclusion of the study, mice were euthanized by CO_2_ inhalation or cervical dislocation. All studies were approved by the San Diego Veterans Administration Medical Center and UCSD Institutional Animal Care and Use Committee (animal-use protocols A17-020 and S99001).

*Antibody Conjugation:* Monoclonal anti-mucin 5AC antibody (Novus Biologicals, Littleton, CO, USA) was conjugated per the manufacturer’s instructions to the near-infrared (NIR) dye IRDye800CW (LI-COR Biosciences, Lincoln, NE, USA) to establish MUC5AC-IR800. For purification, MUC5AC-IR800 was applied to a gel desalting column (Thermo Fisher Scientific, Waltham, MA, USA) to remove excess unbound dye. The final product was stored at 4 °C.

*Patient-derived Liver Metastasis of Pancreatic Cancer:* The patient-derived liver metastasis of pancreatic tumor (*Panc Met*) was obtained under sterile conditions at time of exploratory laparotomy. The patient tumor was obtained with informed consent under the UCSD Institutional Review Board (IRB) protocol number 090401.

*Western Blots*: Normal human pancreas tissue and patient-derived liver metastasis tumor fragments (*Panc Met*) were used to make tumor lysates. Western blotting was performed as previously described [11]. Total protein lysates (80 μg) were loaded on 2% sodium dodecyl sulfate-agarose gels. Proteins were resolved by horizontal electrophoresis for 4 h at 100 V in trisglycine-sodium dodecyl sulfate buffer. The proteins were transferred onto polyvinylidene-difluoride membranes and then blocked in 5% skim milk in phosphate buffered saline (PBS). Membranes were then incubated with a primary antibody (CLH2, Millipore, Burlington, MA, USA) at 4 °C overnight. Next, membranes were washed with PBS/Tween solution three times and incubated with horseradish peroxidase-labeled anti-mouse secondary antibody for 1 h at room temperature. After secondary antibody incubation, membranes were washed again three times with PBS/Tween solution. Protein bands were visualized using a chemiluminescence reagent (Thermo Fisher Scientific, Waltham, MA, USA).

*Xenograft Establishment*: To initially establish *Panc Met* in mouse models, 1 mm^3^ fragments of the patient’s tumor were implanted into the bilateral flanks and shoulders of nude mice. Once subcutaneous tumors grew to approximately 1 cm, subsequent passages were performed by harvesting 1 mm^3^ fragments and implanting them into new mice. For subcutaneous models (*n* = 4), tumors were allowed to grow for ~4 weeks prior to performing imaging studies. To establish patient-derived orthotopic xenograft (PDOX) models, subcutaneously grown tumors were harvested, and 1 mm^3^ fragments were used to establish liver PDOX models (*n* = 3) via the sutureless surgical orthotopic implantation liver technique previously described [15]. In brief, the anesthetized mice had their ventral surface disinfected with 70% ethanol solution. A ~1 cm horizontal incision was made centered over the xyphoid process, and gentle pressure was used to deliver a lobe of the liver extracorporeally. Sharp scissors were used to make a ~3 mm pocket in the liver and pressure was held until hemostasis was achieved. A small tumor fragment was then placed in the pocket, and pressure was held again as necessary for hemostasis. The liver was returned to the peritoneum and a 6-0 vicryl suture (Ethicon Inc., Somerville, NJ, USA) was used to close the incision. Post-operative pain was treated with subcutaneous buprenorphine (25 μL) reconstituted in PBS.

*Antibody-conjugate Administration and Imaging:* MUC5AC-IR800 75 μg was administered via tail vein injection to the subcutaneous models. Subcutaneous models were imaged daily for 4 days (*n* = 4 at each timepoint) using the Pearl Trilogy Small Animal Imaging System (LI-COR Biosciences, Lincoln, NE, USA) with excitation at 800 nm. The Pearl Trilogy Small Animal Imaging System quantified the strength of the NIR signal from the tumor and from the skin, which was used as background for subcutaneous models. A tumor to background ratio (TBR) was calculated by dividing the mean fluorescence intensity (mFI) of the tumor by the mFI of the skin. The mean and standard deviation of the TBRs were calculated for each timepoint. The timing for the orthotopic models was based on the highest TBR from the subcutaneous models. Orthotopic models received MUC5AC-IR800 75 μg via tail vein injection. After 72 h, the mice were euthanized, and a midline laparotomy was made to expose the liver. The Pearl Trilogy Small Animal Imaging System was used to measure the mFI of the tumor and of the liver, which was used as a background. The TBR from each mouse was calculated by dividing the mFI of the tumor by the mFI of the liver. An average TBR and standard deviation were calculated.

*Immunohistochemistry*: Tumor samples were removed en bloc with surrounding tissue at the time of mouse necropsy. Samples were fixed in formalin for at least 72 h prior to being embedded in paraffin and sectioned. Slides were stained with hematoxylin and eosin (H&E) per standard protocols. Immunohistochemistry was performed per standard protocol using a mucin5AC antibody (Abcam ab3469) at a dilution of 1:1000 with secondary anti-mouse HRP polymer (Cell IDx, 2MH-100). Horseradish peroxidase was visualized by a diaminobenzidine (DAB) chromogenic reaction. Interpretation of the histologic slides was performed by an experienced pathologist (MH).

## 3. Results

### 3.1. Patient-Derived Liver Metastasis of Pancreatic Cancer

**Figure 1:**
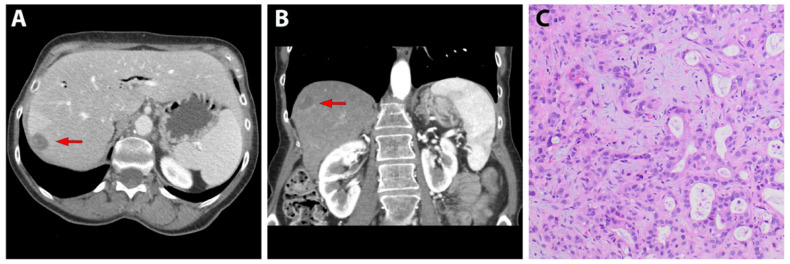
**A** Patient-derived liver metastasis of pancreatic cancer. (**A**) Axial and (**B**) coronal computerized tomography of patient with segment 7 liver metastasis from pancreatic cancer as denoted by a red arrow. (**C**) H&E staining of patient specimen at 20× magnification showing poorly differentiated adenocarcinoma.

An intra-operative biopsy of metastatic pancreatic cancer to the liver was obtained from a 63-year-old female with pancreatic adenocarcinoma. She received neoadjuvant chemotherapy of gemcitabine/abraxane prior to planned pancreaticoduodenectomy. Although imaging and diagnostic laparoscopy did not reveal any metastatic disease, during exploratory laparotomy, a segment 7 liver lesion was noted, and frozen sections confirmed the presence of metastatic disease. Cross-sectional imaging obtained ~3.5 weeks after surgery revealed a 2.2 cm × 1.7 cm hypodense lesion in segment 7 (Figure 1A,B). The pathology demonstrated moderately to poorly-differentiated adenocarcinoma (Figure 1C).

### 3.2. Western Blot of MUC5AC Expression

**Figure 2:**
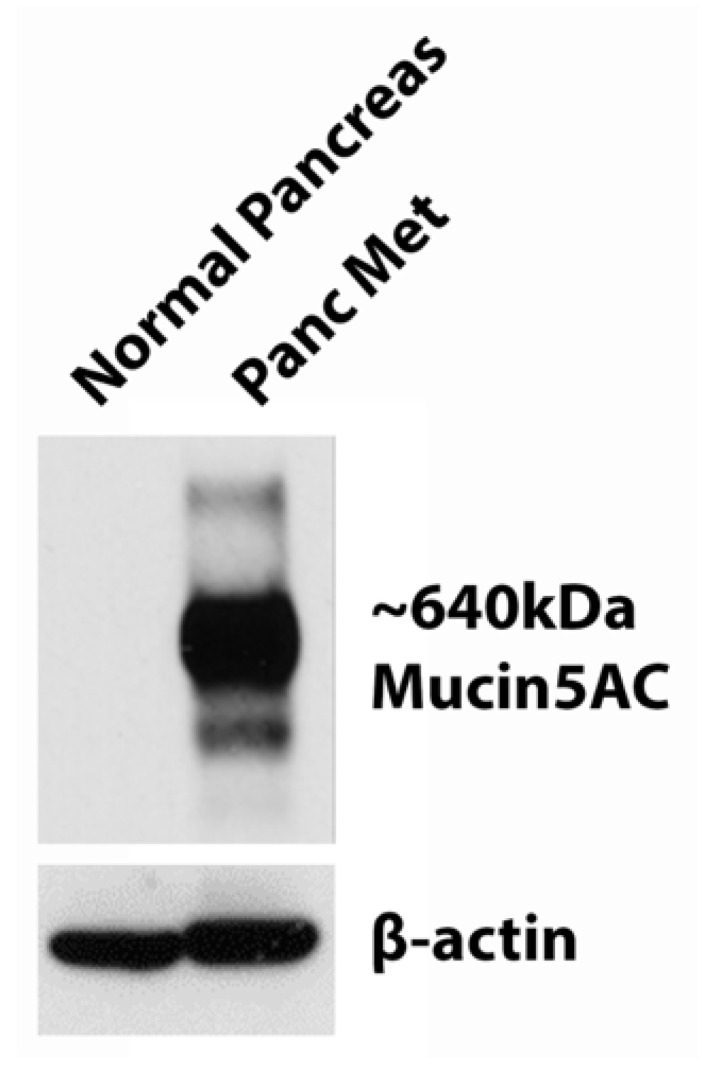
MUC5AC western blotting in normal pancreas and *Panc Met*. Absence of mucin5AC expression in normal human pancreas tissue and high levels of mucin 5AC expression in a patient-derived liver metastasis of pancreatic cancer (*Panc Met*); β-actin as the control.

Western blotting demonstrated no MUC5AC expression in normal pancreatic tissue, while there was a high level of MUC5AC expression in the patient-derived PDAC liver metastasis tumor, *Panc Met* (Figure 2).

### 3.3. Labeling Subcutaneous Tumors with MUC5AC-IR800

In subcutaneous xenograft models, tumor mean fluorescent intensity (mFI) was relatively stable over 72 h at ~0.14 with a slight decrease at 96 h. The background signal was, however, highest at 24 h and decreased on each subsequent day. The peak tumor to background ratio (TBR) was 7.034 ± 3.285 at 72 h (Table 1). Given the highest TBR was achieved at 72 h, this time point was used for the orthotopic models.

### 3.4. Targeting Pancreatic Cancer in the Liver with MUC5AC-IR800

**Figure 3:**
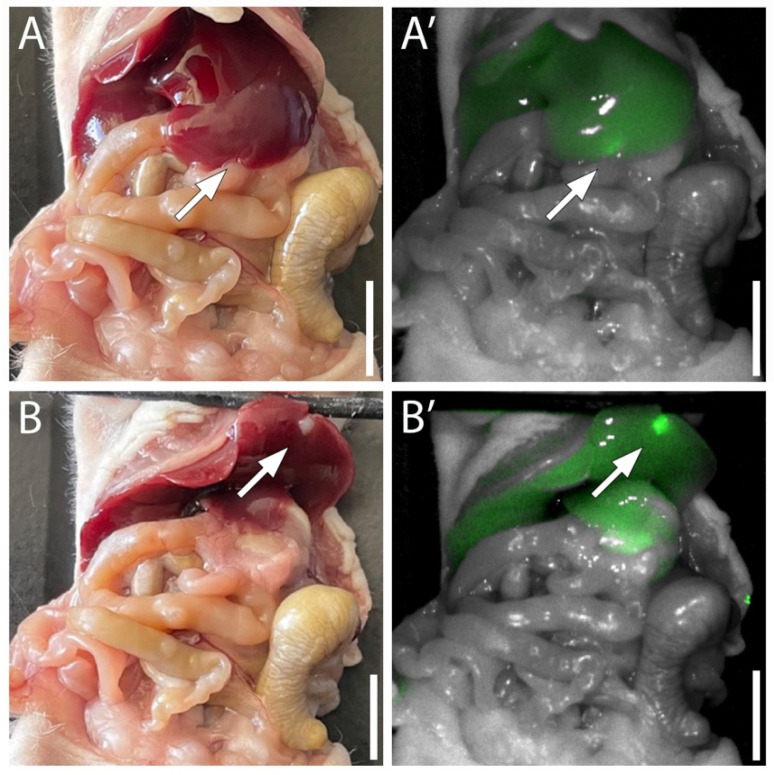
Fluorescence labeling of orthotopic *Panc Met* tumors 72 h after IV injection of MUC5AC-IR800. (**A**) The liver appears normal from the anterior orientation under white light. (**A’**) With fluorescence imaging, a signal can be visualized at the edge of the liver. (**B**,**B’**) Once the liver is retracted cephalad, the fluorescent tumor is clearly visualized. Arrows = tumor. Scale = 1 cm.

*Panc Met* tumors growing in the liver were brightly labeled with MUC5AC-IR800 and clearly distinguishable from the background liver signal (Figure 3). The average tumor mFI was 0.042 (SD ± 0.009) and the average liver mFI was 0.023 (SD ± 0.001). The resulting mean TBR of the *Panc Met* orthotopic liver models was 1.787 (SD ± 0.336).

### 3.5. Immunohistochemistry of Mucin

**Figure 4:**
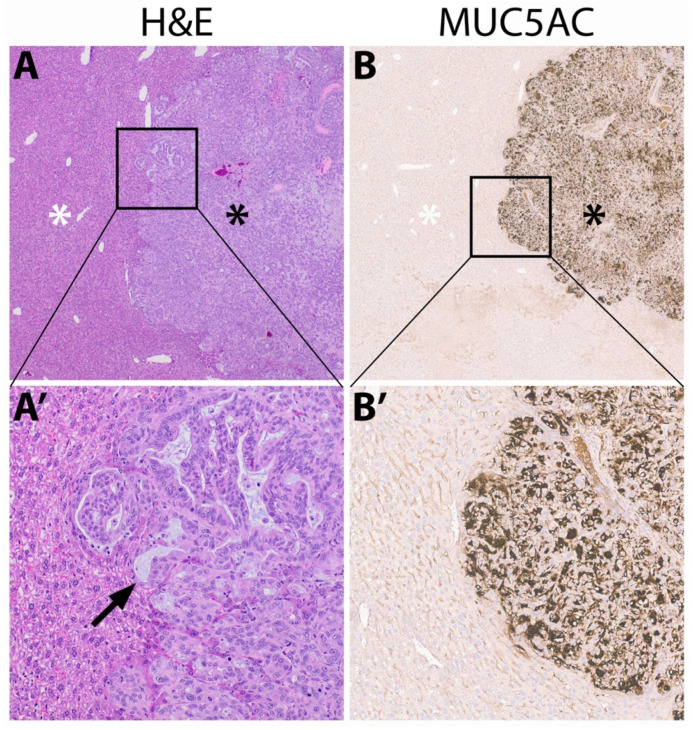
MUC5AC expression in orthotopic *Panc Met* tumors. (**A**) H&E and (**B**) mucin 5AC staining at 2× magnification with adenocarcinoma denoted by a black asterisk, normal liver tissue by a white asterisk. (**A’**) High-power (10×) magnification of H&E staining showing moderately-differentiated adenocarcinoma with few areas of mucin as denoted by a black arrow. (**B’**) High-power (10×) magnification with strong and diffuse cytoplasmic mucin 5AC staining within the tumor.

H&E staining of *Panc Met* orthotopic tumors showed moderately-differentiated adenocarcinoma (Figure 4A,A’). Strong and diffuse cytoplasmic mucin 5AC staining was confined to areas of adenocarcinoma (Figure 4B,B’).

## 4. Discussion

Previously, in a PDOX model of primary pancreatic cancer (*AA1305*), we showed that MUC5AC-IR800 could specifically and brightly label the primary tumor and abdominal wall metastases [11]. However, the previous studies did not address the difficulties and importance of specific labeling of pancreatic cancer liver metastases, as discussed below.

The present study demonstrates that MUC5AC-IR800 can specifically and brightly label metastatic pancreatic cancer implanted in the liver of PDOX mouse models. This is of particular importance given the high background fluorescence signal of the liver noted in prior studies due to the hepatic metabolism of fluorescent antibodies [16]. Previous studies with anti-CEA antibodies used PEGylation of the NIR dye to improve the strength of the signal in the tumor while decreasing hepatic accumulation [17,18,19]. In the present study, we were able to obtain a TBR of 1.737 without PEGylation. For context, clinical trials of fluorescence-guided surgery (FGS) in pancreatic cancer have been able to successfully identify cancerous lesions with TBRs as low as 1.3 [20]. This trial utilized a fluorescent anti-CEA antibody to detect primary pancreatic cancer as well as liver and peritoneal metastases. However, between 40–70% of patients with pancreatic adenocarcinoma do not have an elevated CEA; thus, probes targeting other biomarkers, such as MUC5AC, are necessary [21].

There is growing interest in the use of mucins to label and treat PDAC, and several excellent reviews have been recently published on this subject [12,13,22,23]. Kaur et al. showed that serum MUC5AC levels aided in differentiating PDAC from chronic pancreatitis and benign pancreatic disease [24]. In addition to using fluorescent anti-MUC5AC antibodies to label PDAC, anti-MUC5AC antibodies have been used to image PDAC via PET-CT [25]. Nakata et al. utilized anti-MUC5AC antibodies as a vehicle for therapeutic nuclides in treating PDAC [26]. It should be noted that PDAC is not the only cancer with overexpression of MUC5AC. In addition to PDAC, esophageal, gastric, colon, and ovarian cancer have high expression of MUC5AC, and several of these cancers also have a high rate of metastasis to the liver [23].

Our laboratory has previously discussed the importance of improving the R0 resection rate in pancreatic cancer and the role FGS can play in that endeavor [11]. The present study used a patient-derived pancreatic cancer liver metastasis to demonstrate the labeling of human metastatic pancreatic cancer, a very important tumor site for fluorescence-guided surgery, as well as the identification of such lesions during staging laparoscopy. In addition to the unique challenges of fluorescence imaging in the liver, metastatic pancreatic tumors have notable changes from their primary site: namely their microenvironment, acquired mutations, and the epithelial–mesenchymal transition [27,28]. Previous studies have shown the ability of fluorescence imaging to detect metastatic colorectal tumors at their site of metastasis, but this has not been attempted with metastatic pancreatic cancer [6,8,29]. The ability to identify metastatic pancreatic lesions in the liver is crucial as it can change the surgical plan and potential course of the disease.

The present study was able to utilize a novel tumor model as metastatic pancreatic tumors are rarely resected (metastasis is a contraindication to resection). Given the unique circumstances that allowed us to obtain a pancreatic metastasis from the liver, a more accurate model for this proof-of-concept study was established; conferring the ability to identify and visualize metastatic disease. To the best of our knowledge, no other laboratory has established a patient-derived xenograft mouse model from a metastatic pancreatic tumor. In the present study, we have demonstrated that in addition to bright and specific labeling of the primary tumor, MUC5AC-IR800 is also able to brightly label liver metastases of pancreatic cancer. The ability to detect metastatic tumors is important in directing patients to appropriate therapy, as they would not benefit from an attempt at a curative procedure. Any adjunct to help a surgeon accurately detect even small tumor deposits could spare future patients from long, difficult, and ultimately, futile operations.

Fluorescence detection methods are not widely used in staging laparoscopy in the United States. A surgeon is dependent on visual and tactile clues as well as their own understanding of how and where the disease spreads to detect metastatic tumor deposits. In approximately 23% of cases, staging laparoscopy prior to performing a curative-intent resection for pancreatic cancer will detect previously unappreciated liver metastasis [30]. In nearly all of these cases, the procedure will be aborted. In carefully selected patients, a survival benefit has been shown with the resection of hepatic oligometastatic disease in PDAC [31,32,33,34]. A phase 2 clinical trial is currently underway comparing neoadjuvant therapy and the resection of hepatic oligometastatic pancreatic cancer to historical data [35]. There are some case reports and a small series from Japan describing fluorescence-guided surgery using indocyanine green to perform liver resections [36,37,38].

Limitations of the study include the use of immunocompromised mice, which is necessary to prevent the mouse’s innate immune system from rejecting the xenograft tumor implants. Another limitation is the implantation method of the tumors. The tumors are grafted near the surface of the liver instead of being distributed throughout the liver which is a more accurate depiction of the disease. Fluorescent probes have decreasing signal intensity through tissue and although longer-wavelength probes, such as MUC5AC-IR800, have greater tissue penetration [39], further studies are needed to better define the limits of this technology in terms of tissue penetration, including studies in humans. Clinical trials have shown this technology is translatable into humans [20].

Future studies will include PDOX models with the tumors implanted deeper into the liver parenchyma to be more clinically representative. The present proof-of-concept study indicates the potential for MUC5AC-IR800 to improve detection and possibly resection of hepatic metastasis of pancreatic cancer.

## Figures and Tables

**Table 1:** Fluorescence intensity of subcutaneous tumors targeted with MUC5AC-IR800. Mean fluorescence intensity (mFI) of tumor and background tissue of *Panc Met* subcutaneous tumors after injection of MUC5AC-IR800 (75 µg). Background tissue mFI showed a greater rate of decline every 24 h, resulting in increased tumor to background ratios (TBRs) at later timepoints.

	24 h	48 h	72 h	96 h
Tumor mFI	0.140	0.144	0.143	0.131
Background mFI	0.044	0.031	0.022	0.019
Average TBR (SD)	3.332 (0.884)	4.913 (1.682)	7.034 (3.285)	6.915 (1.880)

## Data Availability

Not applicable.
